# Characterization of a new mutation of mitochondrial ND6 gene in hepatocellular carcinoma and its effects on respiratory complex I

**DOI:** 10.1038/s41598-025-91746-x

**Published:** 2025-03-29

**Authors:** Veronica Bazzani, Deepali L Kundnani, Mara Equisoain Redin, Francesca Agostini, Kirti Chhatlani, Angelo Corso Faini, Jakub Poziemski, Umberto Baccarani, Pawel Siedlecki, Silvia Deaglio, Francesca Storici, Carlo Vascotto

**Affiliations:** 1 IMol Polish Academy of Sciences, Warsaw, 02-247 Poland; 2 School of Biological Sciences, Georgia Institute of Technology, Atlanta, GA 30332 USA; 3 Department of Medicine, University of Udine, Udine, 33100 Italy; 4 Department of Medical Sciences, University of Turin, Turin, 10126 Italy; 5 Immunogenetics and Transplant Biology Unit, Città della Salute e della Scienza Hospital, Turin, 10126 Italy; 6 Institute of Biochemistry and Biophysics, Polish Academy of Sciences, Warsaw, 02-106 Poland

**Keywords:** Hepatocellular carcinoma, Mitochondria, Mitochondrial DNA, ND6 gene mutation, Respiratory complex I assembly, Molecular dynamics simulations., Hepatocellular carcinoma, Cancer genomics, Energy metabolism

## Abstract

Hepatocellular carcinoma (HCC) is the most common form of liver cancer, which often arises from previous liver pathologies such as HBV, HCV, and alcohol abuse. It is typically associated with an enlarged cirrhotic organ. In this study, we analyzed tumor and distal tissues from a patient who underwent liver resection for HCC with no previous pathologies and whose liver showed normal function without signs of cirrhosis. Genetic analysis of mitochondrial DNA (mtDNA) revealed a novel variant of the gene encoding the NADH dehydrogenase subunit 6 (ND6) protein in the tumor tissue. The deletion of a thymidine generated an early stop codon, resulting in a truncated form of the protein (ΔND6) with 50% of the C-terminal primary sequence missing. ND6 is a subunit of the NADH dehydrogenase complex, also known as Complex I, the largest complex in the electron transport chain. Previous studies have linked mtDNA Complex I mutations to mitochondrial disorders and cancer. Through biochemical analyses, we characterized this new mutation and showed that the expression of ΔND6 negatively affects the stability and functionality of Complex I. Data were confirmed by molecular dynamics simulations suggesting conformational rearrangements, overall revealing a leading role of ND6 in the assembly of Complex I.

**Supplementary Information:**

The online version contains supplementary material available at 10.1038/s41598-025-91746-x.

## Introduction

Hepatocellular carcinoma (HCC) is the most common form of primary tumor in the liver and accounts for around 800,000 deaths each year^1^. In 90% of cases, HCC develops in livers affected by chronic diseases, mainly cirrhosis caused by hepatitis B (HBV) and C (HCV), infection, or alcohol abuse^2^. Nowadays, the available treatment options are limited. In addition, determining the best treatment for the patient is still challenging, given the presence of underlying diseases, the burden and extent of HCC, and the different etiologies. Resection and transplantation remain the cornerstones of curative treatment, especially for early-stage disease^3^. Increasing evidence suggests that impairment of the oxidative phosphorylation system (OXPHOS) and the consequent formation of reactive oxygen species (ROS) are closely related to the occurrence and development of HCC^4^. ROS include oxygen and nitrogen free radicals, molecules that are important for cell signaling, cell differentiation, and apoptosis^5^under physiological conditions. An unbalanced production of ROS contributes to oxidative stress, causing aggregation and denaturation of proteins, peroxidation of lipids, and damage to nucleotides. Mitochondrial DNA (mtDNA) is particularly susceptible to ROS-induced damage due to its proximity to the respiratory chain and the limited repair systems available in mitochondria compared to nuclei^6,7^. Several tumor-specific mtDNA somatic mutations have been identified in various cancers and are proposed to contribute to tumorigenesis. However, a thorough characterization of tumoral mtDNA alterations has yet to be achieved due to insufficient paired (tumor/healthy) sample comparisons, the incomplete characterization of some of the proteins encoded by the mitochondrial genome, and the lack of methods for mitochondrial genome engineering^6^. mtDNA encodes for 13 polypeptides of the respiratory chain complexes, 22 tRNA, and 2 rRNA. Seven of these proteins are small hydrophobic subunits of the NADH: ubiquinone-oxidoreductase (ND1, ND2, ND3, ND4, ND4L, ND5, and ND6) and localize in the membrane P-arm of the complex, where they are pivotal for the ubiquinone reduction and proton translocation mechanism. Mitochondrial Complex I is the respiratory chain’s largest and most intricate membrane protein complex. It is able to pump 4 H^+^per NADH consumed across the inner membrane, contributing about 40% of the proton motive force that drives ATP synthase^8^. Even though X-ray crystallography^9^and cryo-EM analyses^10^helped to characterize the structure of Complex I in several species, it is still difficult to deeply investigate the structural and functional role of the ND proteins encoded by the mtDNA^11,12^. The NADH dehydrogenase subunit 6 (ND6) is one of the less studied subunits. Nonetheless, its role in Complex I has been investigated in relation to Complex I assembly, stability, and function, suggesting a pivotal role of the protein in the Complex I structure^13,14^. In this study, we had the rare opportunity to compare the tumor and distal tissues of a 68-year-old Caucasian male affected by HCC but with no previous history of alcohol abuse and negative for both HBV and HCV. The patient was diagnosed with a large HCC in the right lobe, while the rest of his liver retained normal function, a condition that occurs in only 10% of HCC cases. Genetic analysis of tumor mtDNA revealed the presence of a new mutation: a deletion of thymidine at 70% of the mtDNA molecules (m.14423 A>-), resulting in a frameshift in the gene coding for the protein ND6, with the consequent creation of an early stop codon. After confirming the presence of a truncated form of ND6 (ΔND6) in mitochondria isolated from the tumoral sample, we investigated the effect of ΔND6 on the OXPHOS, revealing the negative outcome on Complex I stability and activity without any evident impact on the levels of the other complexes of the respiratory chain. Molecular dynamics simulations suggested that the loss of alpha helices in the C-term of the protein contributes to the conformational rearrangements, leading to the instability of the complex. Overall, our study shows that a small hydrophobic protein like ND6 significantly impacts Complex I and, consequently, the whole maintenance of the mitochondrial respiratory chain. The determining role of ND6 can be exploited in the future by identifying new therapies targeting ND6 to perturb OXPHOS and eventually interfere with tumor development.

## Results

### Genetic characterization of HCC tissues revealed a new mutation of the mtDNA-encoded ND6 gene

A genetic analysis was performed on samples obtained from a patient affected by HCC with a very atypical condition: he had no history of alcoholism and tested negative for HCV and HBV. Nevertheless, he developed a well-differentiated HCC (G1) with a maximum diameter of 9.5 cm, absent microvascular invasion, resulting in a pathological staging of pT1, N0, and M0. Liver function was normal without signs and symptoms of portal hypertension (Child-Pugh score A-6 and MELD 7). The patient was thereafter scheduled for surgical resection. His preoperative α-fetoprotein (AFP) was 4.8 ng/ml. He underwent an eventful open right hepatectomy in 2015; during surgery, the liver was macroscopically normal without cirrhosis. The patient has been followed up yearly since the last follow-up in April 2022, when he tested negative for HCC recurrence, with normal liver function and an AFP of 4.6 ng/ml. The genomic DNA was analyzed in Next-Generation Sequencing to identify possible mutations linked to the development of HCC. Table 1 lists the alterations identified in the genomic DNA of both the tumoral and distal liver tissue, suggesting a predisposition of the patient to develop the tumor, but without pointing to any specific causative alteration. Unique mutations were detected in four genes in the tumoral sample (MUC22, EPPK1, PRB4, and APEX1). MUC22 and PRB4 have never been associated with HCC before. EPPK1 has a mild association with HCC, while APEX1 has also been extensively studied in liver carcinoma by our laboratory^15^. Sanger sequencing was performed to confirm the presence of these mutations, but the results were negative. We decided, therefore, to focus our attention on mitochondrial DNA. mtDNA sequencing revealed the presence of specific alterations exclusively in the tumoral mtDNA (Table 2). Heteroplasmy levels resulted particularly high in gene encoding COI and ND6. The mutation on COI m.6570G > T was described previously in a patient with cardiomyopathy^17^, while ND6 m.14,423 A>- (and the consequent m.14425 C > T) have never been described before.

**Table 1:** Genetic background of patient ND6-WT. The table lists the alterations identified in distal and tumor samples from patient ND6-WT. Abbreviation: chrom = chromosome; ref = references.

Gene	Chrom	Mutation	Description	Somatic Status	Ref
AXIN1	16	c.2187–1869 A > G	Unknown – Intronic mutation	Confirmed somatic variant	-
	16	c.1254 + 17G > A	Unknown – Intronic mutation	Reported in another cancer sample as somatic	^16^
	16	c.−19 C > A	Unknown – Intronic mutation	Reported in another cancer sample as somatic	^16^
TSC2	16	c.3814 + 932G > A	Unknown – Intronic mutation	Confirmed somatic variant	-
	16	c.3751 + 226G > A	Unknown – Intronic mutation	Confirmed somatic variant	-
KMT2C	7	c.2763 A > G	Substitution – Coding silent	Confirmed somatic variant	-
	7	c.1012 + 13 C > T	Unknown – Intronic mutation	Confirmed somatic variant	-
PTPRB	12	c.2189-88T > C	Unknown – Intronic mutation	Confirmed somatic variant	-
ATM	11	c.6347 + 131T > C	Unknown – Intronic mutation	Confirmed somatic variant	-
KMT2D	12	c.7479G > T	Substitution – Coding silent	Confirmed somatic variant	-

**Table 2:** Mitochondrial DNA mutations identified exclusively in the tumoral sample of patient ND6-WT. Abbreviation: rsid = reference SNP cluster ID; chrom = chromosome; ref = reference allele; alt = alternative, non-reference allele; aa = amino acid; AD ALT % = Allelic depth (aligned sequencing read count) a percentage of the alternative allele with mutation.

Gene	rsID	Chrom	Mutation	Condon change	REF AA	ALT AA	Mutation Type	AD ALT %	Mapping quality	Impact	Ref
ND2	-	MT	m.4813T > C	gTc/gCc	Val	Ala	SNP	18.128	60	MODERATE	-
CO1	rs386828988	MT	m.6570G > T	Gcc/Tcc	Ala	Ser	SNP	75.484	60	MODERATE	^17^
ND6	-	MT	m.14,423 A>-	Tca/-ca	Ser	Gln	INDEL	70.739	59.9	HIGH	-
ND6	-	MT	m.14,425 C > T	ggG/ggA	Pro	Ser	SNP	70.830	59.9	LOW	-

### Expression of ΔND6 affects complex I stability and activity

**Fig. 1:**
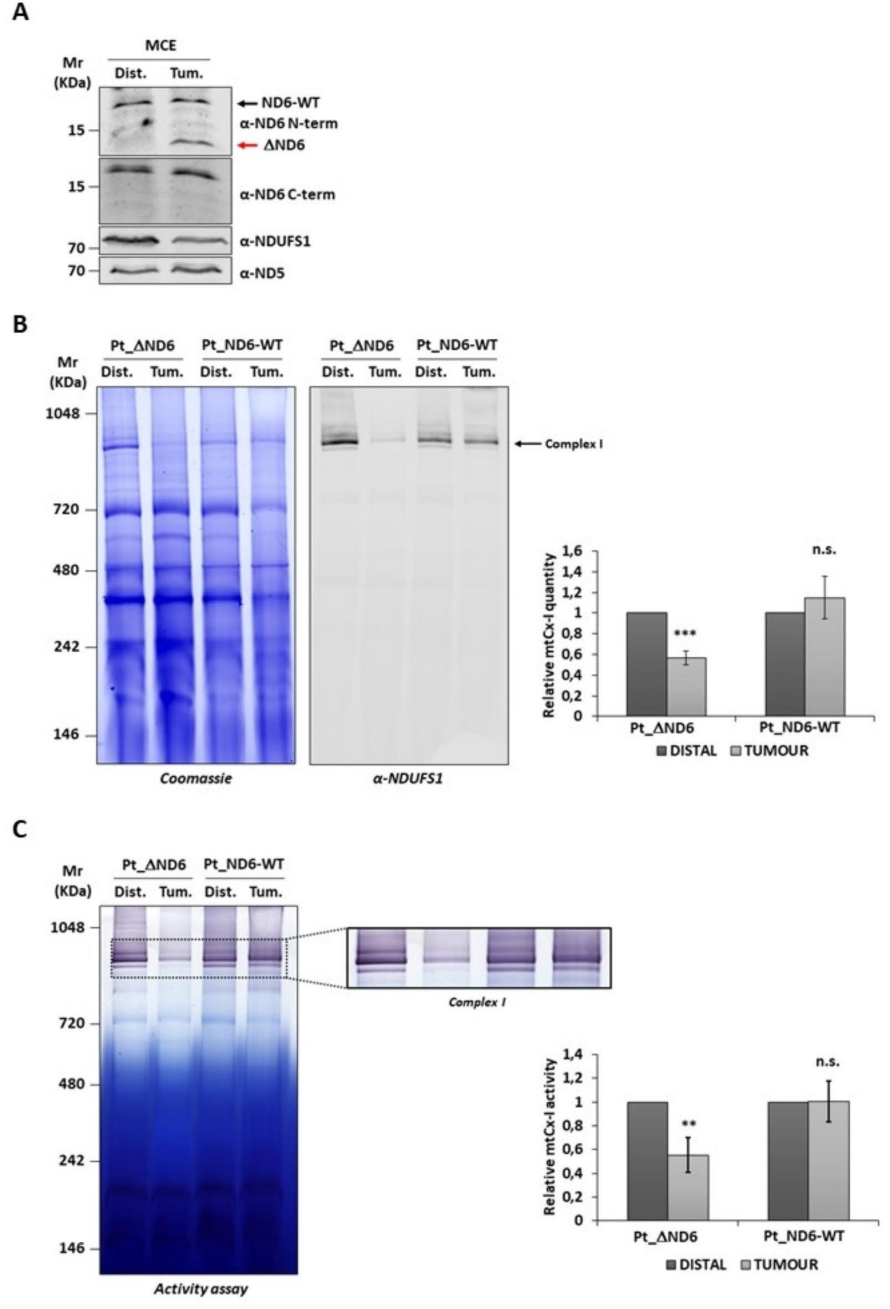
Assembly and activity of Complex I are reduced in tumor tissue of case-study patients expressing ΔND6 form. **(A)** Western blot analyses of 40 µg of the distal (Dist.) and tumoral (Tum.) isolated mitochondria. ND6 protein was detected using two different anti-ND6 antibodies: one recognizing an epitope located in the N-terminal part of the protein (ND6 N-term), which detects both the wild-type (ND6) (black arrow) and the truncated (ΔND6) forms (red arrow). A second antibody recognizes an epitope at the C-terminus of the protein (ND6 C-term), which is not present in the truncated form. NDUFS1 and ND5 antibodies were used as loading controls. **(B)** Representative images of BN-PAGE gel followed by Coomassie staining (l*eft*) and Western blot (*right*) with anti-NDUFS1 antibody of the distal and tumoral liver-isolated mitochondria of the case-study patient (Pt_ΔND6) and a control patient (Pt_ND6-WT) without ND6 mutation. Expression of ΔND6 resulted in a significant reduction of Complex I in tumor mitochondria. In the graph is reported the BN-PAGE densitometric analysis of Complex I (mtCx-I) from three independent replicates (***: *p* ≤ 0.001). **(C)** Representative image of Complex I in-gel activity assay of the distal and tumoral liver-isolated mitochondria of the case-study patient (Pt_ΔND6) and a control patient (Pt_ND6-WT) without ND6 mutation. In the graph is reported the densitometric analysis of mtCx-I activity from three independent replicates (**: *p* ≤ 0.01).

To establish whether the mutated ΔND6 form was expressed, 40 µg of mitochondrial protein extracts (MCE) from the distal and tumor tissues were separated on SDS-PAGE, immunoblotted, and incubated with two anti-ND6 primary antibodies. The α-ND6 C-term antibody, which recognizes an epitope at the protein’s C-terminal, revealed the full-length protein’s presence in both the distal and tumor samples. The α-ND6 N-term antibody, which specifically recognizes an epitope at the N-terminal, besides revealing the signal of the full-length protein (ND6-WT), showed only in the tumor sample the presence of a lower molecular weight band compatible with the truncated ΔND6 protein (Fig. 1A and Supplementary S1-A). To evaluate the effect of the expression of the truncated form on the stability of the respiratory Complex I, 100 µg of mitochondria of the study patient (Pt_ΔND6) were separated on native BN-PAGE, and the gel was stained with Coomassie (Fig. 1B and Supplementary S1-B, *left*) or blotted and immunorecognized with Complex I protein NDUFS1 (Fig. 1B and Supplementary S1-B, *right*). As a control, the same amount of mitochondria isolated from the distal and tumor liver tissues of a patient not carrying the m.14,423 A>- mutation (Pt_ND6-WT) were also analyzed. Densitometry analysis of Complex I showed a significant reduction of Complex I in the tumor tissue concerning the distal one due to the expression of ΔND6 (56% ± 6.5%). On the contrary, no difference was observed in the tissues of the WT patient. The densitometry analysis of Complex III and V showed no significant difference between the distal and tumor samples, confirming that the expression of the mutant ΔND6 exclusively affects the stability of Complex I (Fig. 1B and Supplementary S2). Next, we evaluated whether the mutation compromised the activity of the complex. An in-gel activity assay of Complex I was performed by incubating the BN-PAGE gel with a solution containing NADH and nitrotetrazolium blue chloride, which reacts, changing color only in the presence of an active Complex I (Fig. 1C and Supplementary S1-C). The activity was normalized to the amount of stable Complex I detected by BN-PAGE, with Coomassie staining shown in Fig. 1B. The densitometry revealed a significant decrease in the Complex I activity in the mitochondria of the tumor tissue (55% ± 14%) compared to the distal one. Conversely, in agreement with the results of the previous analysis, mitochondria from the patient with ND6-WT showed comparable Complex I levels between the distal and tumor samples (Fig. 1C).

### Molecular dynamics simulations confirm the instability of ΔND6 within the respiratory complex

**Fig. 2:**
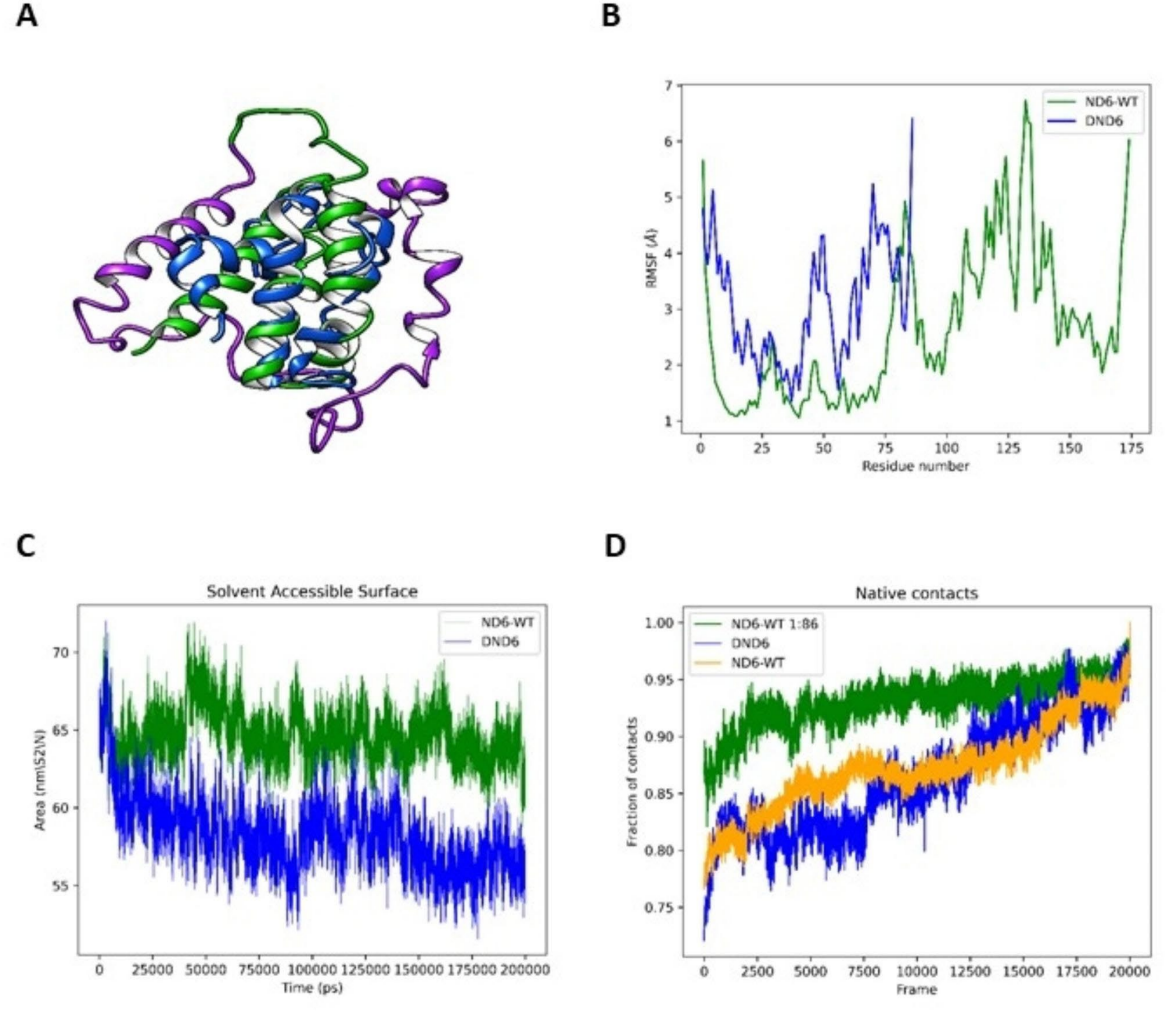
Molecular dynamics simulations of ND6-WT and ΔND6 reveal conformational changes upon loss of the C-terminal region. **(A)** Ribbon representations of the ND6-WT (green), ΔND6 (blue), and the missing C-terminal fragment (violet). **(B)** Comparison of the Residual Mean Square Fluctuation (RMSF) indicates increased movement in the N-terminal region of ΔND6 compared to WT. **(C)** Solvent Accessible Surface Area (SASA) analysis reveals that the N-terminal region of ΔND6 adopts a more compact conformation than ND6-WT. **(D)** Analysis of native contacts in ΔND6 shows that only a quarter of the original ND6-WT molecular contacts are present; however, the retained native contacts remain stable. Overall, the MDS suggests that ΔND6 undergoes conformational rearrangements, resulting in a relatively stable, non-native structure that resists unfolding and degradation.

Biochemical analysis on isolated mitochondria proved that the truncated ΔND6 mutant form is expressed in the tumor tissue and negatively impacts the stability and activity of Complex I. Molecular dynamics simulations were performed to support these experimental evidences and confirm that ND6 conformation and stability within the complex are affected by the loss of the C-term (Fig. 2A). Residual Mean Square Fluctuation (RMSF) analysis showed elevated movement of ΔND6 in the N-terminal region, which is conformationally stable in the wild-type protein, suggesting a spatial rearrangement or possible partial unfolding compared to the WT (Fig. 2B). Solvent Accessible Surface Area (SASA) analysis suggests that the truncated form of ND6 adopts a more compact conformation compared to the corresponding wild-type ND6 (Fig. 2C). Low fluctuations in the solvent-accessible area hint at conformational rearrangements occurring in ΔND6, rather than an unfolding event caused by the truncation of the C-terminus. To assess the extent of conformational rearrangements, we compared the preservation of native contacts in truncated and wild-type ND6 structures. Figure 2D shows that ΔND6 does not retain approximately one-quarter of the original contacts (defined as Cα atoms less than 7 Å apart), diverging from the original model structure during simulation. Despite ΔND6 losing the largest fraction of its original contacts compared to wild-type ND6, the retained fraction of native contacts remains stable in the later stages of the simulation. The molecular dynamic simulation (MDS) experiments suggest that ΔND6 undergoes conformational rearrangements rather than misfolding or unfolding events leading to protein degradation. As such, the modified and relatively stable truncated conformation of ΔND6 may enable it to interact negatively with Complex I via its N-terminal region.

### Assembly of complex I is affected by the presence of ΔND6

**Fig. 3:**
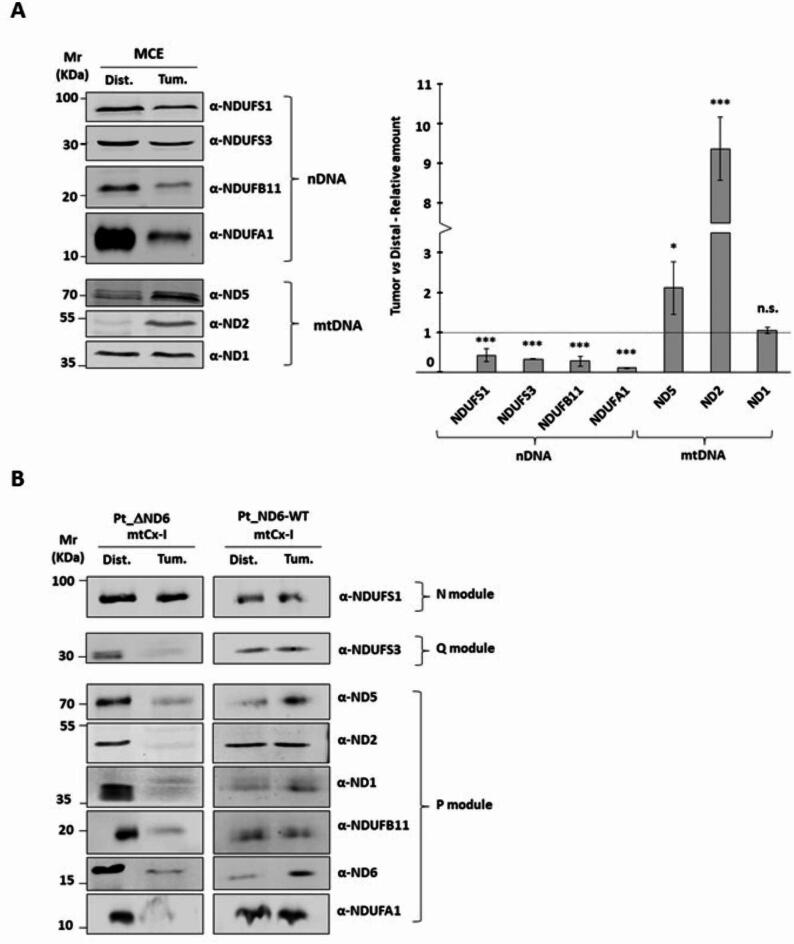
mtDNA-encoded Complex I proteins are overexpressed but not efficiently assembled in tumor mitochondria. **(A)** Representative Western blot analysis of 40 µg of mitochondrial extract from distal and tumor tissues separated on SDS-PAGE. The expression of nuclear DNA (nDNA)-encoded proteins reduces the mitochondrial DNA-encoded proteins that are upregulated in tumor mitochondria. In the graph are reported the means and standard deviations of three independent replicates expressed as relative to distal tissue (*** *p* ≤ 0.001; ** *p* ≤ 0.01; * *p* ≤ 0.05). **(B)** Normalized amounts of BN-PAGE resolved Complex I were isolated and separated on SDS-PAGE to evaluate the levels of mtDNA-encoded proteins for both Pt_ND6-WT and Pt_ΔND6.

Biochemical and bioinformatics analyses support the hypothesis that ΔND6 is affected by conformational changes that influence protein integration within the complex. Hence, we decided to investigate the Complex I subunits’ expression and their composition in the tumoral and distal isolated mitochondria in more detail. 40 µg of mitochondria were separated on SDS-PAGE, and the levels of four nuclear (NDUFS1, NDUFS3, NDUFB11, NDUFA1) and three mitochondrial (ND1, ND2, ND5) encoded proteins belonging to Complex I were evaluated (Fig. 3A and Supplementary S3). Nuclear-encoded proteins decreased in the tumoral samples, while the mitochondrial-encoded protein levels were either unchanged (ND1) or significantly increased (ND2, ND5). We deepened our analysis by performing a 2D Blue Native/SDS-PAGE to explore the levels of subunits integrated into Complex I. The analysis was performed on both Pt_ΔND6 and Pt_ND6-WT. The amount of tumoral Complex I loaded on SDS-PAGE was adjusted to normalize the samples using NDUFS1, a nDNA-encoded subunit belonging to the last module included in the holo-complex as a reference. Pt_ΔND6 showed a similar level of NDUFS1 in distal and tumoral Complex I when four times more Complex I from the HCC sample was loaded on the gel. The level of NDUFS1 of Pt_ND6-WT was similar when loading the same amount of Complex I from distal and tumor. Surprisingly, in Pt_ΔND6, even if NDUFS1 levels are comparable between distal and tumor (Fig. 3B and Supplementary S4), it is evident the lower presence in Complex I of all analyzed encoded subunits belongs to the Q and P modules. This suggests that even if the full Complex I is assembled, it is not as stable as in the healthy tissue of Pt_ND6-WT, where all the subunits show similar levels or an increase in the tumoral sample.

## Discussion

HCC represents 90% of all known liver malignancies and, accounting for more than 800,000 deaths worldwide in 2020, has a mortality rate second only to lung cancer^18^. Therapies available can be classified as curative or palliative. The selection of one approach over the other is mainly based on physical tumor parameters (size, number of lesions, and extent of extrahepatic invasion), the complexity of comorbidity, and the patient biochemical profile. Systemic therapies based on molecular and cellular approaches have emerged in the last decade. Nevertheless, the prognosis of advanced HCC remains poor, with a five-year survival rate that does not go above 20–40% at best^19^. The subject of this study is a patient who developed HCC in the context of a healthy liver, providing the rare opportunity to perform analyses comparing healthy and tumor tissues from the same donor. While genetic analyses revealed the presence of gene mutations that could be considered predisposing factors for HCC, sequencing of mtDNA unveiled a new mutation of the tumor’s mitochondrial gene ND6 (MT-ND6). Mutations in MT-ND6 have been associated with several rare mitochondrial disorders and cancers^20–22^. The m.14484T > C variant is responsible for about 15% of all cases of Leber hereditary optic neuropathy^23^. Several publications have also reported mutations in MT-ND6 linked to Leigh syndrome^24–26^. In addition, alterations have been described in several cancers, mainly in the metastatic context^27–30^. Unfortunately, the molecular mechanisms involved in these pathologies remain unclear, as well as how the same mutation can, in some cases, cause varied signs and symptoms^24^.

**Fig. 4:**
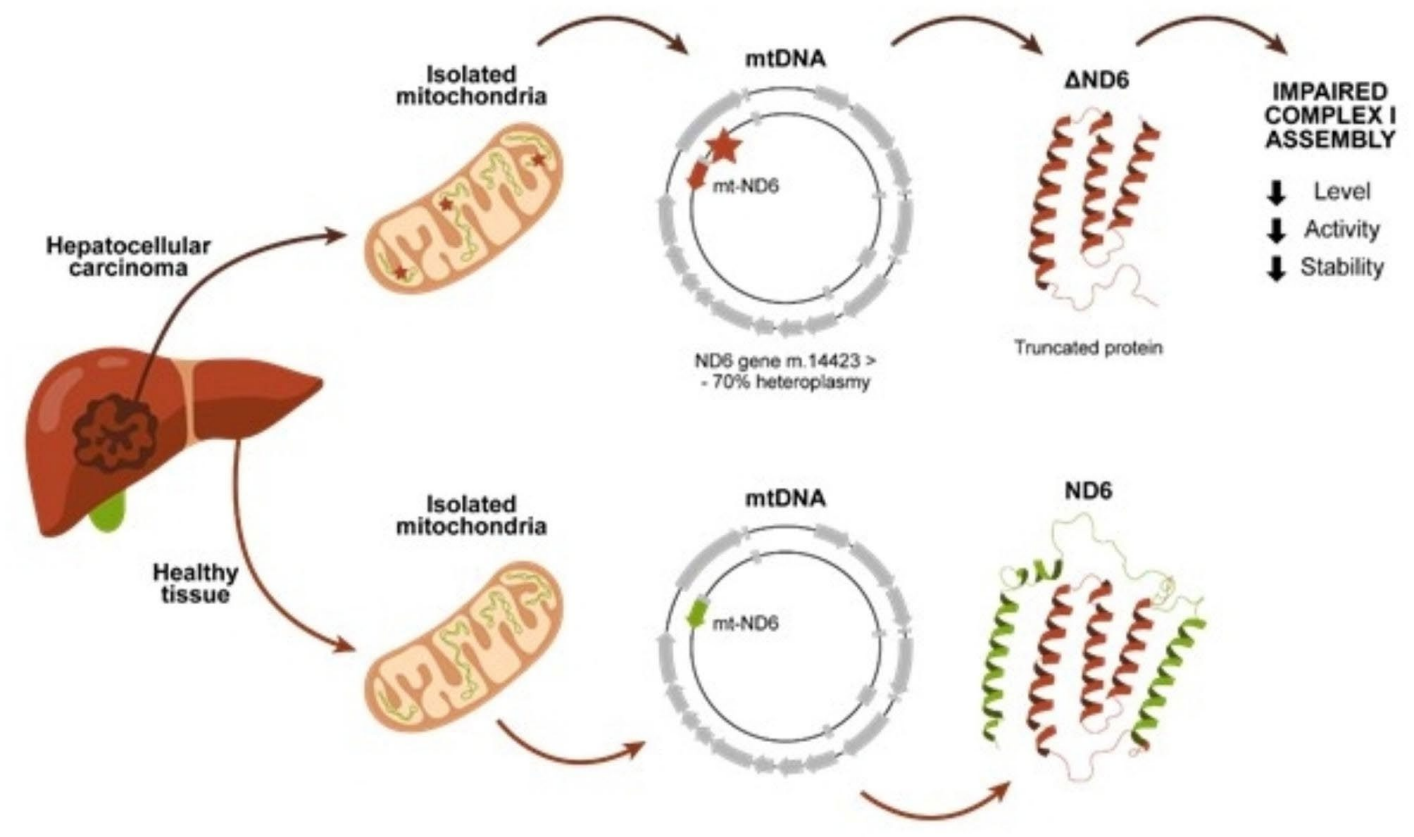
Patient ΔND6 developed HCC in the right liver lobe without any evident triggering factor. The mtDNA genetic analysis revealed a mutation on the ND6 gene present only in the tumor with a heteroplasmy level of around 70%. The m.14,423 > - mutation leads to an early stop codon and, consequently, to a truncated form of the protein, which loses the C-terminal alpha helices (green). The presence of a truncated form of ND6 negatively affects Complex I assembly, decreasing the levels of the holo-complex and its activity.

ND6 is a subunit of Complex I, located at the junction between the P and the Q module. In this study, we characterized a new mutation of MT-ND6, which determines the loss of the C-terminal of the protein (ΔND6) (Fig. 4). The truncated form of the protein encoded in the HCC lesion loses 3 alpha helices involved in the interaction with the Q module. Laube et al. showed in their 2022 study of *Chaetomium thermophilum*the pivotal function of ND6 in creating the E-channel, whose role is to allow the flow of electrons in Complex I^31^. It is possible to speculate that the whole assembly of Complex I is affected in a heteroplasmic situation, where 70% of the mtDNA carries the truncated form of ND6. To test this hypothesis, we first confirmed the expression of ΔND6 in the HCC tumoral mass and performed molecular dynamics simulations to underline the intrinsic instability of the truncated ND6 form. The MDS supports the hypothesis that the expression of this mutant interferes with the interaction with other subunits of the respiratory Complex I, negatively affecting its stability and, consequently, its activity.

The assembly of Complex I is the most elaborate of all the OXPHOS complexes. Complex I contains 45 subunits, whose amount and interactions must be finely regulated. The accepted model describing the formation of the holo-complex is based on the production and assembly of modules in a series of parallel, coordinated steps^32,33^. Each module merges with the other to create a fully functional Complex I. ND6 is integrated into the ND2 module at an early stage: the deficiency of the WT ND6 can easily cause a stalemate in the formation of the module. As a matter of fact, when investigating the relative composition of Complex I nuclear and mitochondrial codified subunits present in isolated mitochondria, we noticed that the latter were either equally expressed (ND1) or upregulated (ND2, ND5) in the tumor. On the opposite side, nDNA-encoded subunits were significantly less expressed in the tumor compared to the healthy control (Fig. 3A). In human cells, nDNA-encoded subunits of each OXPHOS complex are not synthesized in stoichiometric balance, and orphaned OXPHOS proteins are rapidly degraded by protein quality control pathways^34–37^. Our observations in HCC support the hypothesis that the degradation works at the level of the nDNA-encoded subunits, possibly regulating the levels of proteins coming from the cytosol to match the availability of fully functional ND6. Consistent with the decreased levels of nDNA-encoded proteins, we observed a decrease in the levels of Complex I on BN-PAGE (Fig. 1B). Intriguingly, when we evaluate the activity of Complex I in gel normalizing the amount of complex between the distal and tumor samples, we observe that the capacity of the holo-complex to process the nitrotetrazolium blue chloride in Formazan is reduced in the tumor (Fig. 1C). This reduced activity could be explained by a problem in the function and/or in the structure of the complex, somehow related to the presence of ΔND6 in the pool of subunits that can assemble into Complex I. For this reason, we decided to check whether the subunits forming the tumor’s holo-complex were normally integrated into the complex. Unexpectedly, when measuring the single subunit composition in the complex via 2D analysis, we observed a significantly decreased amount of subunits belonging to the P and Q modules of Complex I. This evidence is specific to the patient carrying the deletion on ND6. Indeed, the same analysis performed on Pt_ND6-WT shows that the subunits of the P and Q modules have similar levels between distal and tumoral Complex I (Fig. 3B). The 2D analysis confirms that the tumoral Complex I of Pt_ΔND6 is not fully functional. Unfortunately, the lack of established methodologies for editing the mitochondrial genome prevents further investigation to better clarify the loss of activity of Complex I. Nevertheless, our findings suggest a leading role of ND6 in the assembly and functionality of the respiratory Complex I. Moreover, our data support the hypothesis that the assembly of Complex I is strictly regulated by the fluxes of cytosolic subunits into mitochondria^37^. Finally, in contrast with the MT-ND6 mutations characterized in cancer until now^27,30,38,39^, we did not observe any relationship between Complex I alterations and metastasis, suggesting that ND6 could be part of the *oncojanus* genes, a category of genes that differently impact tumor progression depending on both their mutation load and type, making ND6 an interesting gene for future therapeutic investigations^40^.

## Methods

### Human hepatocellular carcinoma and adjacent non-tumor tissue specimens

Samples of paired HCC (tumor) and adjacent non-tumor liver tissues (distal) were collected from the Department of Medicine, General Surgery, and Transplantation of the University of Udine, Udine, Italy. Samples carrying the ND6 mutation were obtained from a 68-year-old Caucasian male diagnosed with a large HCC located in the right liver lobe during a routine ultrasound (Pt_ΔND6). The patient had not received any local or systemic anticancer treatments before the surgery, had no history of alcohol intake, and tested negative for HCV and HBV. Samples expressing ND6 WT were obtained from a 72-year-old Caucasian female diagnosed with HCC (Pt_ND6-WT). In both cases, the diagnosis of HCC was performed by preoperative computed tomography (CT) imaging scan. Hepatic serology, α-fetoprotein (AFP), and routine laboratory assessment of liver and renal function were also performed. After hospital discharge, the patients were followed up and monitored for tumor recurrence by monthly assessments of serum AFP and by CT or magnetic resonance imaging (MRI) every 3–6 months according to international guidelines.

### Approval, accordance and informed consent

This study was approved by the Unique Regional Ethics Committee on August 24, 2019, Protocol number 18,659, and written informed consent was obtained from the patients. All methods were performed in accordance with the relevant guidelines and regulations.

### Mitochondria isolation from human HCC tissue specimens

Fresh samples were finely minced, suspended in 5 mL of Isolation Buffer (IB) [10 mM Tris/MOPS, 1 mM EGTA/Tris, 200 mM Sucrose], and homogenized. Then, samples were centrifuged at 70 x g for 3 min to remove non-homogenized tissue. The supernatant was further centrifuged at 600 x g for 10 min to separate nuclear (pellet) and mitochondrial (supernatant) fractions. Mitochondria were centrifuged at 7000 x g for 10 min, washed once in IB buffer, and resuspended in IB buffer. Mitochondrial protein extracts were quantified using Bradford protein assay reagent.

### Whole exon sequencing and MtDNA sequencing

Genomic DNA was extracted using the QIAmp DNA Mini Kit (Qiagen). Briefly, samples were resuspended in 180 µl of Buffer ATL complete with 20 µl of proteinase K and incubated at 56 °C until the samples were completely lysed. 200 µl of Buffer AL and 200 µl of ethanol (96–100%) were added, and the solution was incubated for 5 min at room temperature. Lysates were loaded into the QIAamp MinElute column and centrifuged at 6000 x g for 1 min. Two washes were performed with solutions AW1 and AW2. Elution was performed at 6000 x g for 1 min. Eurofins performed next-generation sequencing.

To confirm the alteration identified with the NGS analysis, the following primers were designed to perform Sanger sequencing on genes APEX1 and PRB4: APEX1_FW: TAGCTTGCTATCCACTGCCT; APEX1_REV: TGCAGTAATTCCCCGAAGCC; PRB4_FW: AGATCGGGCACTTCGGGACT; PRB4_REV: GTGCATTTCCCCATTTAGCTCCA.

mtDNA was extracted from isolated mitochondria using the NucleoSpin Plasmid kit (Macherey-Nagel), following the manufacturer’s instructions. Paired-end DNA sequencing of the mitochondrial genome was performed using HiSeq X (Illumina). The data generated from sequencing was trimmed using Trimgalore (v0.6.7) to filter out readings with quality lower than 15 and shorter than 50 bp in length. The high-quality data was then aligned to the hg38 chrM reference genome using BWA-MEM (v0.7.17). Information merging of pair-end readings was performed using Samtools (v1.7). Two different methods were used for variant calling from GATK (v4.2.2.0): HaplotypeCaller, which is widely used for pooled samples but provides both germline and somatic variants, and Mutect2, widely used to call somatic variants in mitochondria as it is more sensitive to variants present in a small percentage of readings. Variants found by both tools were selected to filter out somatic variants present in a significant percentage of readings in each sample library. A customized code was used to calculate the percentage of readings containing the mutants at their respective locations and to merge the percentages of every mutant for patient samples. Mutants found exclusively in either distal or tumor liver tissue biopsy samples were listed with the percentages of aligned readings containing that mutant. The annotations for Reference SNP cluster ID (rsID) were added using bcftools from dbSNP (v151) to report known vs. novel variants. Associated gene, codon, and amino acid changes; disease pathways for the gene, mutation impact, and known clinical significance were annotated using the Ensemble Variant Effector Predictor (VEPv104). After determining the ND6 mutant as interesting, the consensus sequence of the ND6 gene in distal and tumor samples was derived using bcftools, and the ND6 sequence from the reference was derived using bcftools. Since the ND6 sense strand is on the reverse strand, the ND6 open reading frames for samples and references were reverse complemented and translated using Expasy.

### Western blot analysis

40 µg of mitochondrial extract from each sample were separated into 15% SDS-PAGE. The proteins were then transferred into a nitrocellulose membrane (Sartorious). Membranes were saturated by incubation with 5% BSA or 5% non-fat dry milk, depending on the antibody, in TBS-T [1X TBS supplemented with 0.1% Tween 20] for 1 h. Blots were incubated with the primary antibody overnight at 4 °C [α-ND6: 1:500 polyclonal (MBS8518686, MyBioSource), 1:500 polyclonal (NBP1-70650, Novus Biologicals); α-NDUFS1: 1:500 monoclonal (ab169540, Abcam); α-NDUFS3: 1:10000 monoclonal (68066-1-Ig, Proteintech); α-NDUFB11: 1:2000 polyclonal (16720-1-AP, Proteintech); α-NDUFA1: 1: 500 monoclonal (ab176563, Abcam); α-ND1: 1:1000 polyclonal (19703-1-AP, Proteintech); α-ND2: 1:1000 polyclonal (19704-1-AP, Proteintech); α-ND5: 1:500 polyclonal (ab138136, Abcam); α-OxPhos: 1:1000 monoclonal (ab110411, Abcam)]. Membranes were washed three times for 5 min with TBS-T, incubated with the secondary antibody (IRDye 800, IRDye 680 LI-COR Biosciences) for 2 h, and rewashed three times for 5 min with TBS-T. Blots were acquired using the Odyssey DLx scanner (LI-COR Biosciences), and densitometric analysis was performed with ImageStudio software (LI-COR Biosciences). Western blots of BN-PAGE gels were performed with the Trans-Blot Turbo Transfer System (BIO-RAD), using PVDF membranes (Trans-Blot Turbo Mini 0.2 μm PVDF Transfer Packs, BIO-RAD). After blotting, membranes were fixed in 8% acetic acid for 15 min, destained in methanol, and rinsed in deionized water. Finally, membranes were saturated by incubation with 5% non-fat dry milk in TBS-T, incubated with primary and secondary antibodies, and acquired as described.

### BN-PAGE analysis and complex I activity

To test Complex I stability, 500 µg of mitochondria isolated from distal and tumor samples were lysed in 1X NativePAGE Sample Buffer (Life Technologies) with 6 mg/mg of mitochondria of Digitonin (Calbiochem) in a final volume of 50 µL. Samples were incubated on ice for 15 min and then centrifuged at 20,000 x g, 4 °C for 30 min. 10 µL of supernatant of each sample was then separated on BN-PAGE. 2 µL of NativePAGE 5% G-250 Sample Additive (Life Technologies) was added to each sample before electrophoresis, which was performed using NativePAGE Novex Bis-Tris Gel 4–16% (Life Technologies). Running conditions were optimized to efficiently resolve the Complex I band. Electrophoresis was carried out at 4 °C at 150 V constant for 1 h, then the voltage was increased to 250 V constant for 4 h. During the first 4 h of electrophoresis, 1X NativePAGE Dark Blue Cathode Buffer (Life Technologies) was used, while during the last 1 h, it was substituted with 1X NativePAGE Anode Buffer (Life Technologies). After electrophoresis, gels were either stained with Comassie R-250, processed for the detection of Complex I *in-gel* activity, or for the 2D analysis of Complex I subunits. For the Coomassie R-250 staining, gels were placed in Fix solution [40% methanol, 10% acetic acid] and microwaved for 45 s; then they were incubated on an orbital shaker for 15 min at RT; Fix Solution was decanted, Destain Solution [8% acetic acid] was added, and gels were microwaved for 45 s; gels were finally incubated on an orbital shaker for 2 h at RT. For the *in-gel* activity assay, BN-PAGE gels were incubated with the Activity Solution [2.5 mg/mL nitrotetrazolium blue chloride, 0.1 mg/mL NADH, in Tris/HCl 5 mM pH 7.4] for 2 h at RT on an orbital shaker. Subsequently, gels were fixed in Fix Solution. For the 2D assay, Complex I was cut from the BN-PAGE gel, loaded onto a 15% SDS-PAGE gel, and WB was performed as described above. All the acquisitions were performed using the Odyssey DLx scanner (LI-COR Biosciences), and densitometric analysis was performed with ImageStudio software (LI-COR Biosciences).

### Molecular dynamics simulation

Two CryoEM structures (5XTC, 5XTD) together with the Alphafold model (AF-Q6XBD3-F1-v4) were compared. All simulations were performed using the GROMACS suite (version 2022). Proteins were immersed in a TIP3P cubic water box with a distance of at least 1 nm between the protein and the edge of the simulation box. Simulations were run with the AMBER99SB force field with periodic boundary conditions. Energy minimization was performed using steepest descent minimization with a maximum of 50,000 steps and a maximum force of 10 kJ/mol. Next, the system was equilibrated in the NVT ensemble for 100 ps with modified Berendsen thermostat heating; as a result, a temperature of 300 K was reached. After this step, 100 ps NPT equilibration was performed using the Parrinello-Rahman barostat with 1 atm pressure. At the end, a 200 ns production molecular dynamics simulation was performed.

### Statistical analysis

Statistical analysis was performed using Microsoft Excel. The students’ t-test was used to compare the two groups. *P-*values of less than 0.05 were considered significant, while values less than 0.01 or lower were considered highly significant.

## Electronic supplementary material

Below is the link to the electronic supplementary material.

Supplementary Material 1

## Data Availability

For whole exome sequencing, UCSC genome browser sessions with uploaded sequence tracks have been made available at https://genome.ucsc.edu/s/cvascotto/hg38_ND6_data. For mtDNA, DNA-seq data are available in NCBI’s Sequence Read Archive, as SRR23726624 and SRR23726649 for distal and tumor liver tissue. UCSC genome browser sessions with uploaded sequence tracks have been made available at https://genome.ucsc.edu/s/dlk_browse/Patient8_ND6. The mtDNA sequencing data analysis, variant calling, and ND6 gene consensus sequence generation source code is published on Zenodo: https://doi.org/10.5281/zenodo.13926899.
