# Supplementary material for: Characterization of a new mutation of mitochondrial ND6 gene in hepatocellular carcinoma and its effects on respiratory complex I
Source: Sci Rep. 2025 Mar 29;15:10925. doi: 10.1038/s41598-025-91746-x (PMC11954883; doi:10.1038/s41598-025-91746-x)
Supplement: Supplementary file 1 — Supplementary Material 1 [file 41598_2025_91746_MOESM1_ESM.docx]

**Supplementary Figure S1**

**A**

**Mr**

**(KDa)**

**
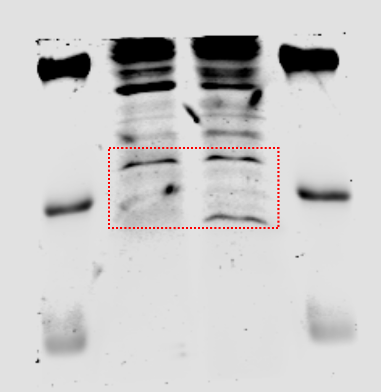
**

**15**

**25**

**α - ND6 N-term**

**
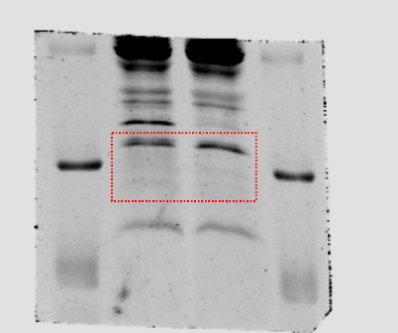
**

**10**

**25**

**15**

**α - ND6 C-term**

**
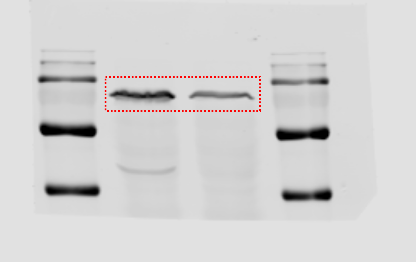
**

**70**

**55**

**α - NDUFS1**

**35**

**
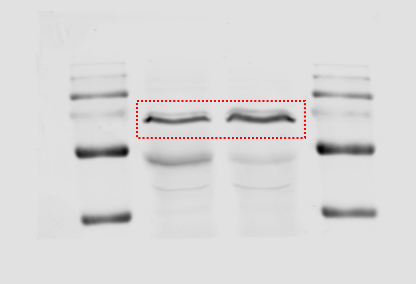
**

**100**

**70**

**55**

**α - ND5**

**35**

**
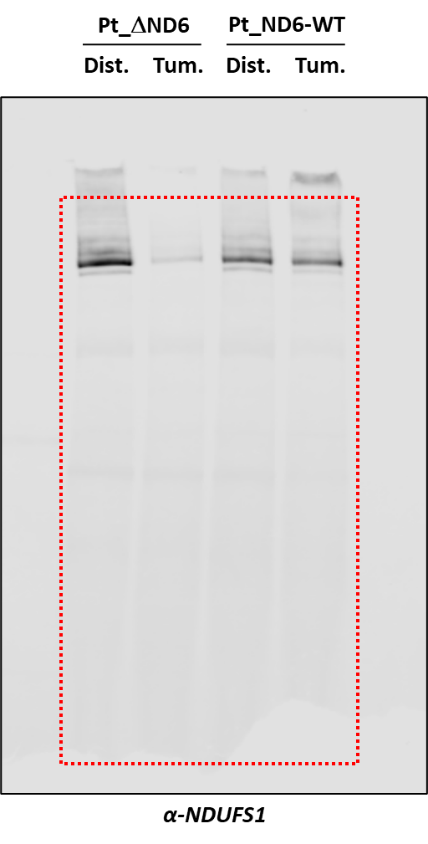

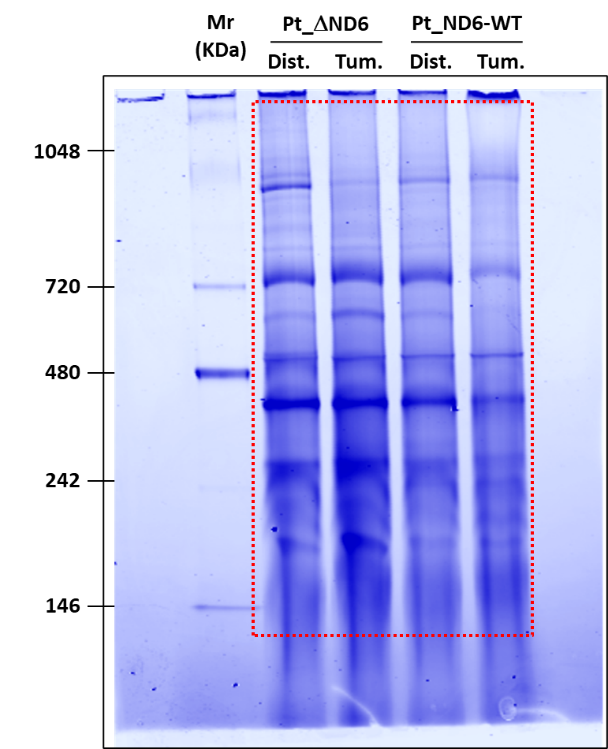
**

**B**

**
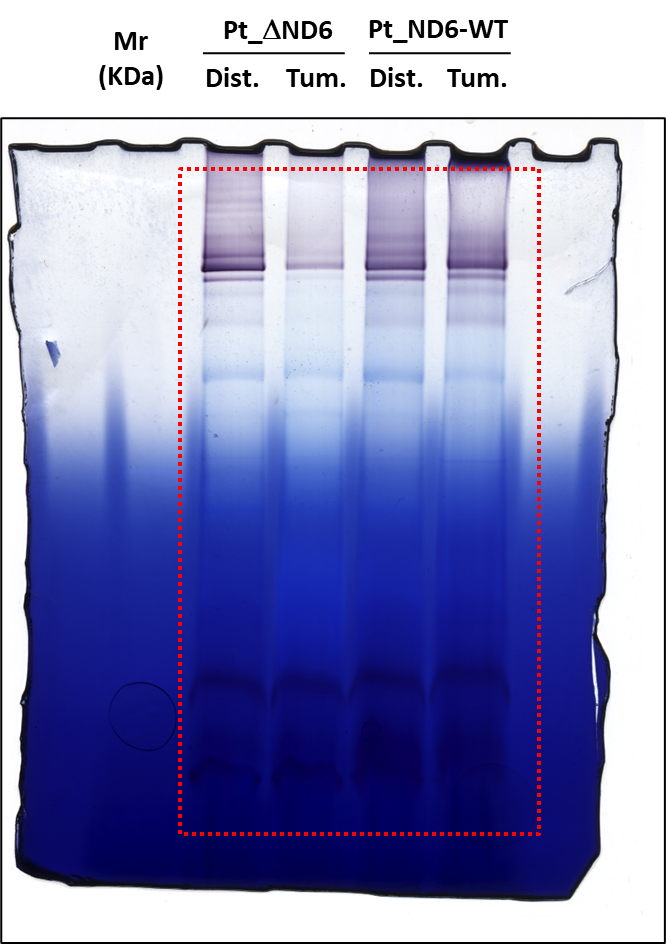
**

**C**

**Supplementary Figure S1:** **(A)** Uncropped Western blot images from the blots shown in Figure 1A. **(B)** Uncropped images of BN-PAGE gel followed by Coomassie staining (*left*) and Western blot (*right*) with anti-NDUFS1 shown in Figure 1B. **(C)** Uncropped image of the Complex I in-gel activity assay reported in Figure 1C. The red boxes outline the areas presented in Figure 1.

**Supplementary Figure S2**

**A**


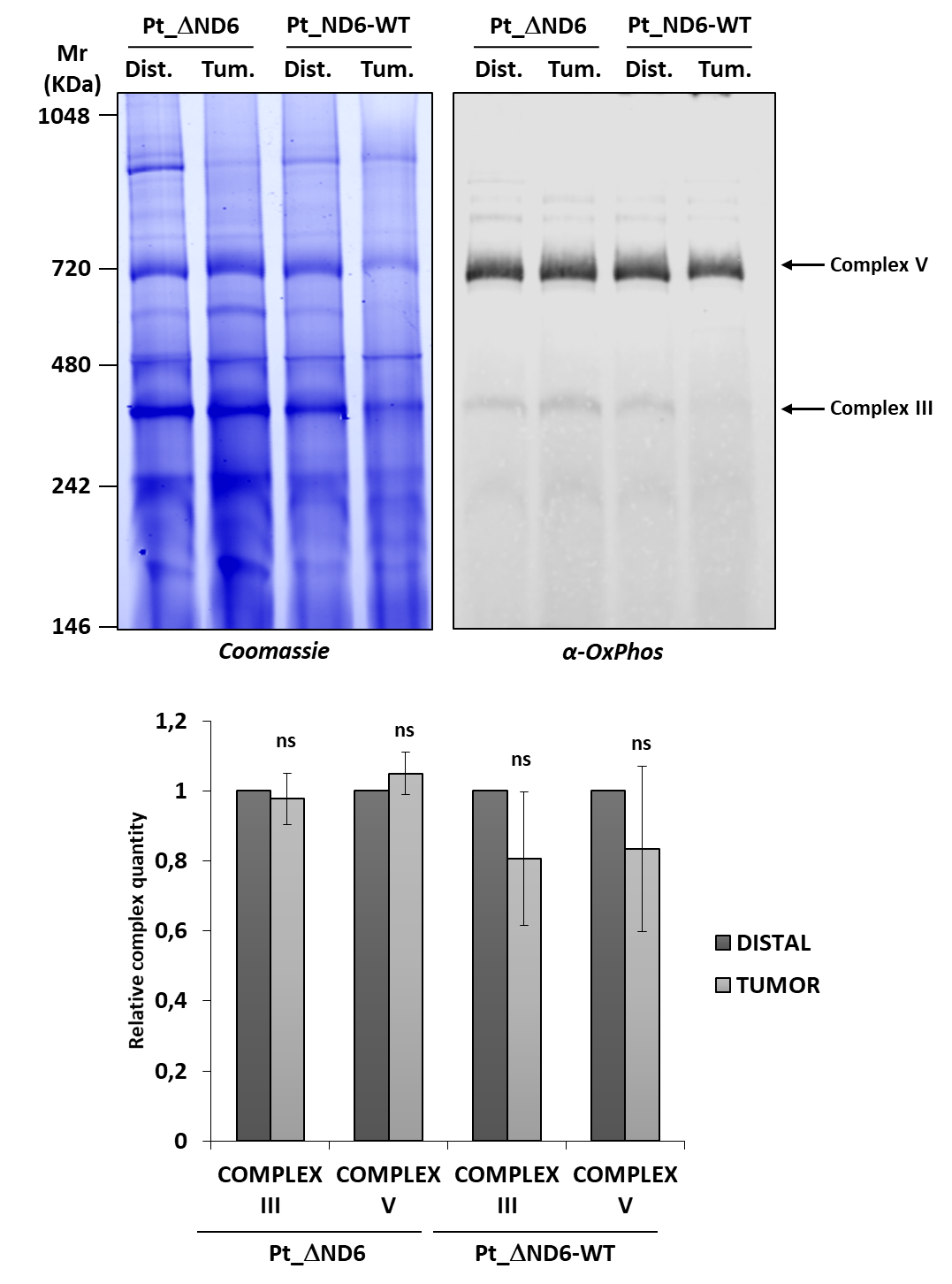


**B**

**
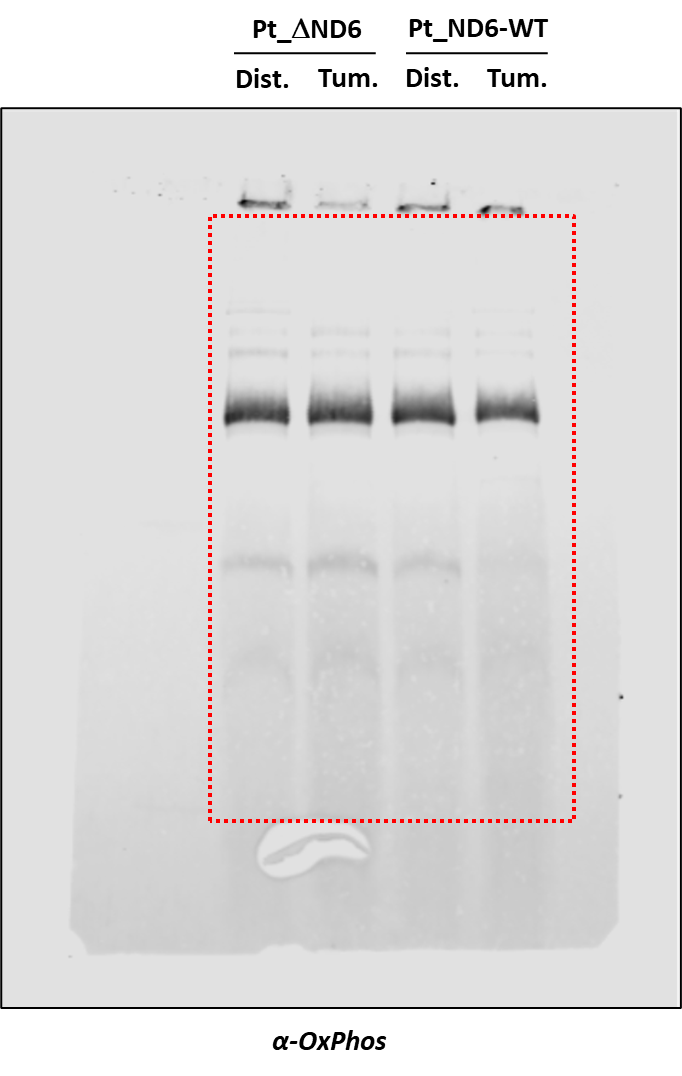

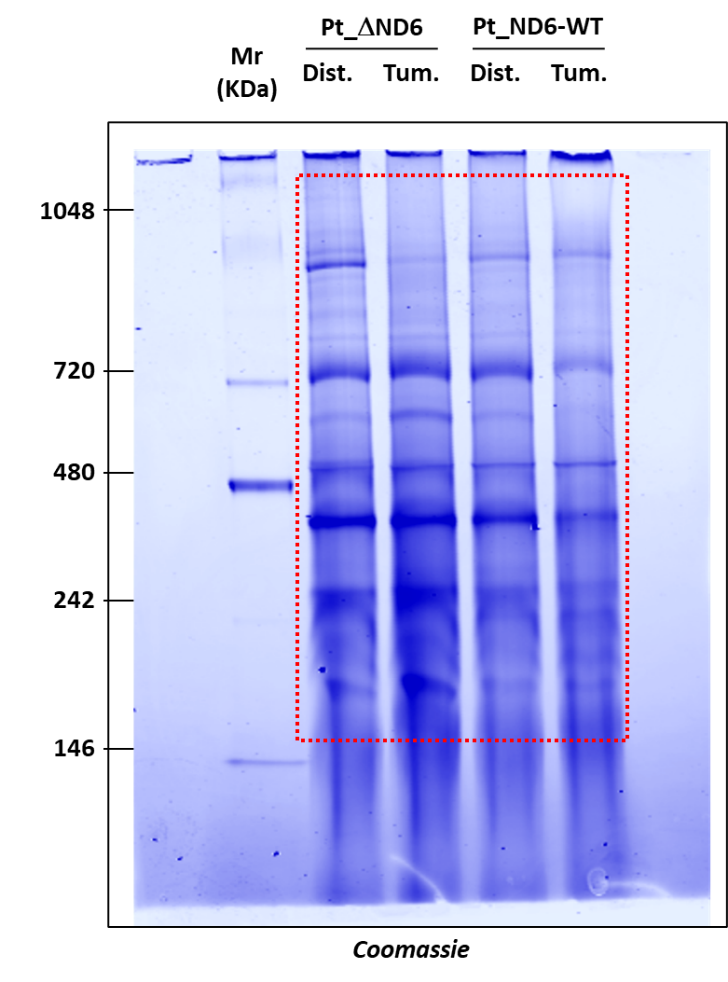
**

**C**

**Supplementary Figure S2: (A)** Representative images of BN-PAGE gel followed by Coomassie staining (*left*) and Western blot (*right*) with anti-OxPhos antibody of the distal and tumoral liver isolated mitochondria of the case-study patient (Pt_ΔND6) and a control patient (Pt_ND6-WT) without ND6 mutation. Complex III and V are equally expressed in both patients. **(B)** In the graph is reported the BN-PAGE densitometric analysis of Complex III and V from three independent replicates. **(C)** Uncropped images are reported. The red boxes outline the areas presented in Supplementary Figure S2A.

**Supplementary Figure S3**

**
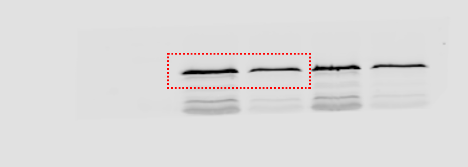
**

**α - NDUFS1**

**
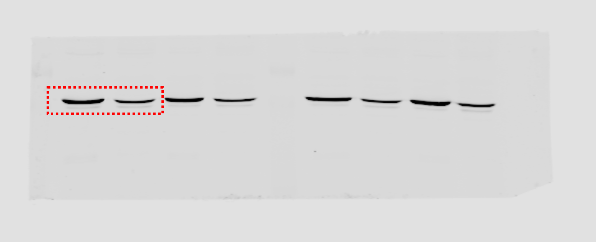
**

**α - NDUFS3**

**
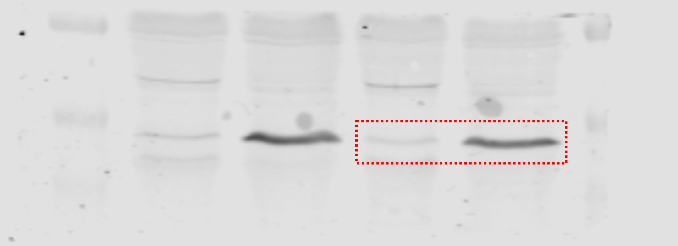

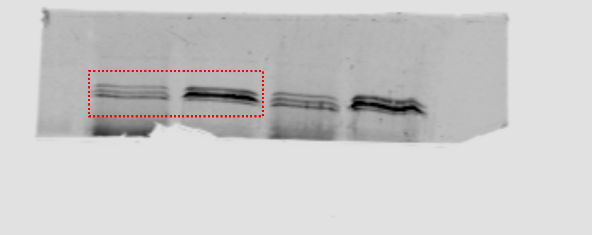

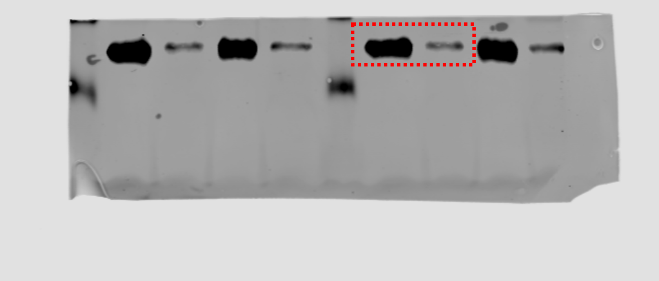

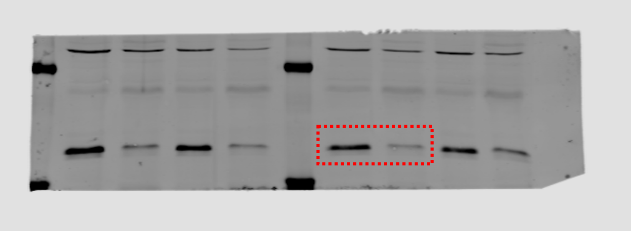
**

**α - ND1**

**α - ND2**

**α - ND5**

**α - NDUFA1**

**α - NDUFB11**

**25**

**15**

**15**

**10**

**70**

**55**

**
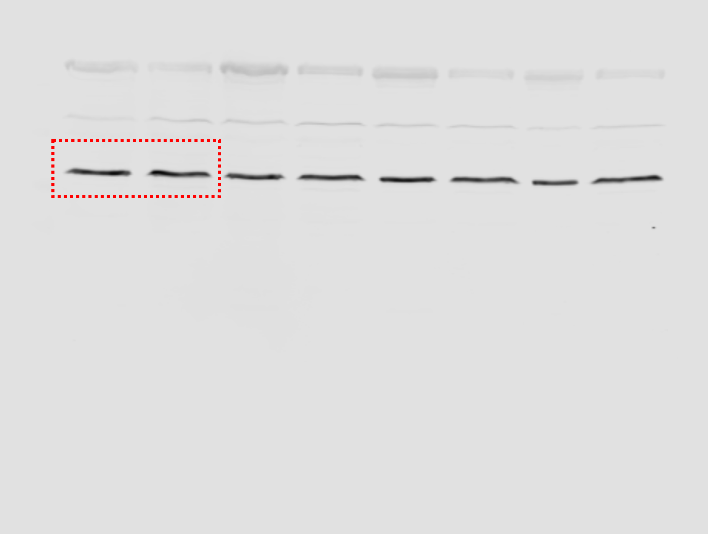
**

**Supplementary Figure S3:** Uncropped Western blot images from the blots shown in Figure 3. The red boxes outline the areas presented in Figure 3A.

**Supplementary Figure S4**

**
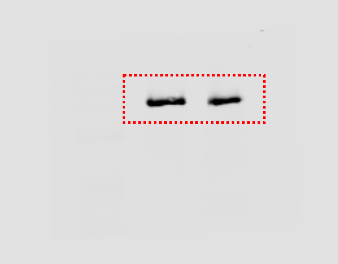

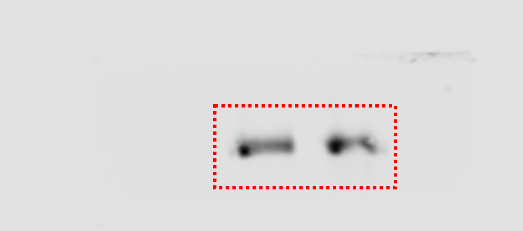
**

**α - NDUFS1**

**α - NDUFS1**

**
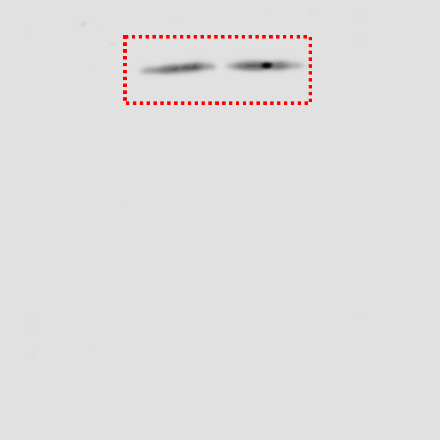
**

**
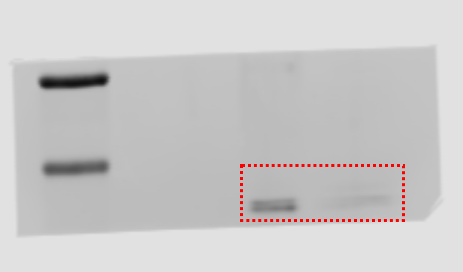
**

**70**

**α - NDUFS3**

**α - NDUFS3**

**55**

**
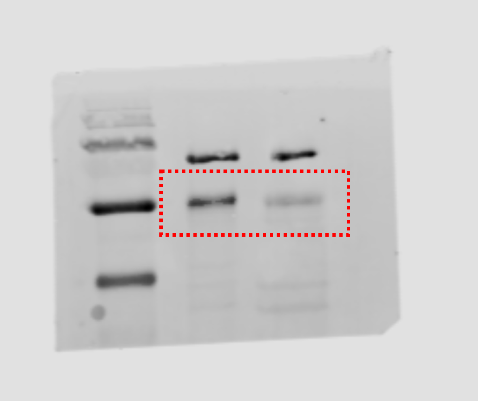
**

**
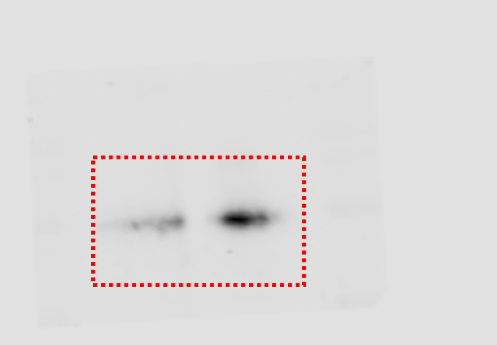
**

**70**

**α - ND5**

**α - ND5**

**55**

**
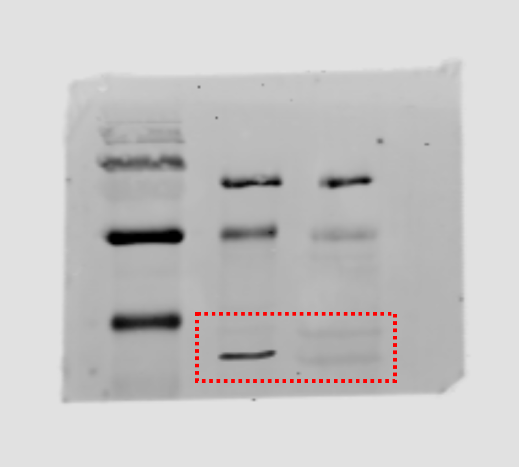

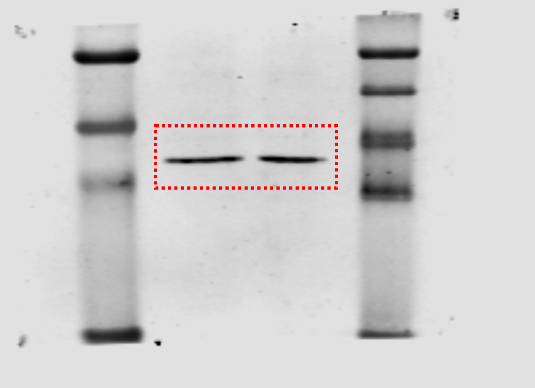
**

**70**

**55**

**α - ND2**

**35**

**70**

**α - ND2**

**25**

**55**

**
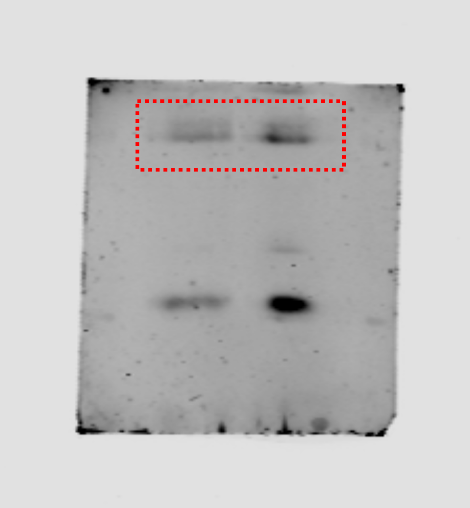
**

**
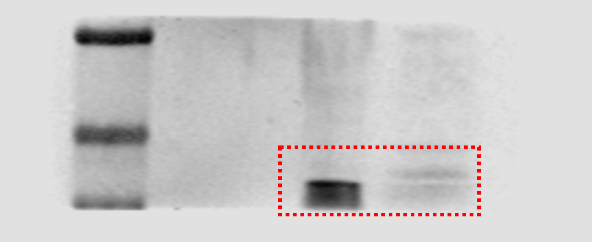
**

**70**

**55**

**α - ND1**

**α - ND1**

**35**

**
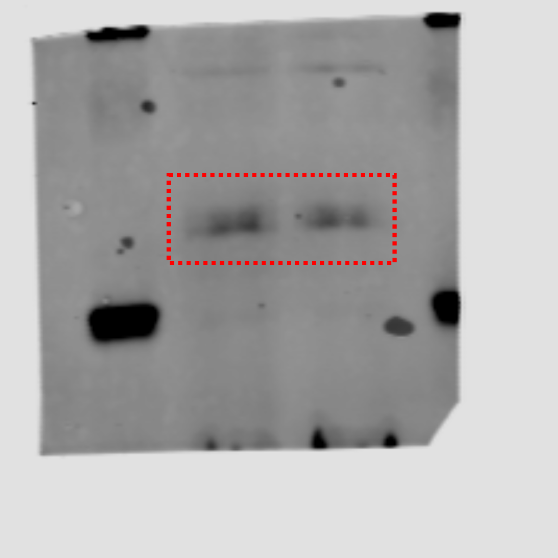

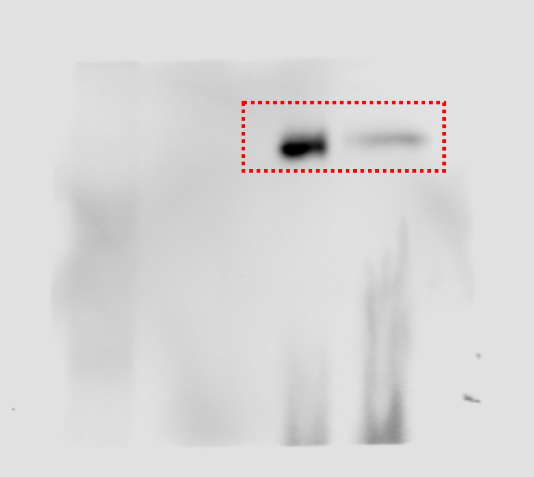
**

**α - NDUFB11**

**α - NDUFB11**

**15**


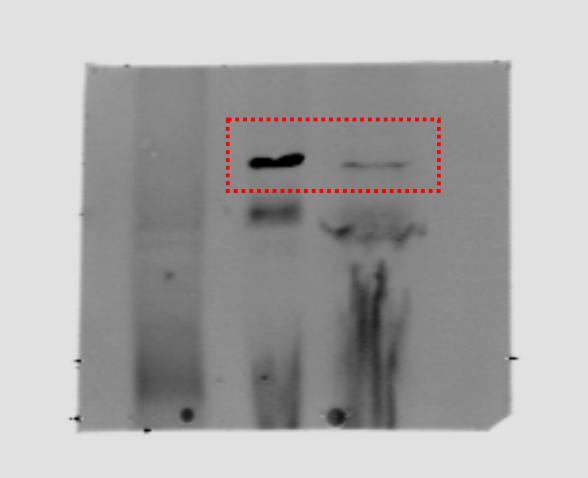


**α - ND6**


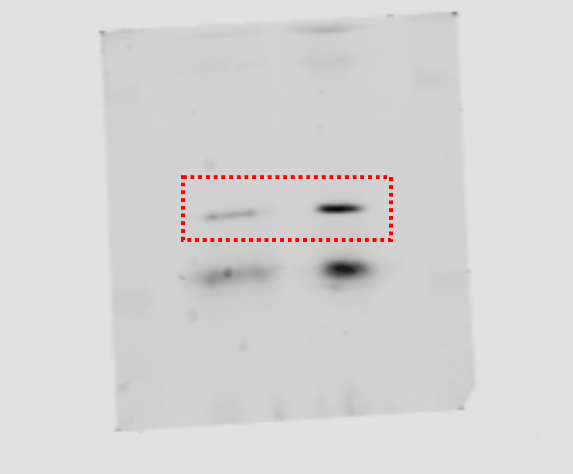


**α - ND6**


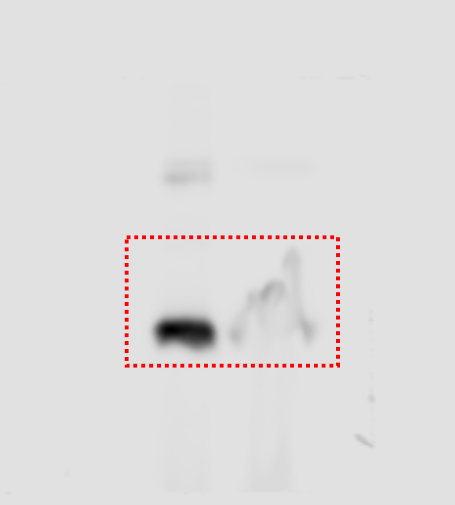


**α - NDUFA1**


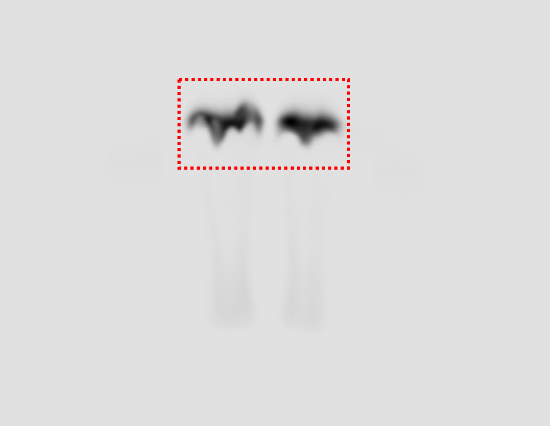


**α - NDUFA1**

**Supplementary Figure S4:** Uncropped Western blot images from the blots shown in Figure 3. The red boxes outline the areas presented in Figure 3B.
